# NF-Protocadherin Regulates Retinal Ganglion Cell Axon Behaviour in the Developing Visual System

**DOI:** 10.1371/journal.pone.0141290

**Published:** 2015-10-21

**Authors:** Louis C. Leung, William A. Harris, Christine E. Holt, Michael Piper

**Affiliations:** 1 Department of Physiology, Development and Neuroscience, University of Cambridge, Cambridge, UK, CB2 3DY, United Kingdom; 2 The School of Biomedical Sciences and the Queensland Brain Institute, The University of Queensland, Brisbane, QLD 4072, Australia; NIH/NEI, UNITED STATES

## Abstract

Cell adhesion molecules play a central role in mediating axonal tract development within the nascent nervous system. NF-protocadherin (NFPC), a member of the non-clustered protocadherin family, has been shown to regulate retinal ganglion cell (RGC) axon and dendrite initiation, as well as influencing axonal navigation within the mid-optic tract. However, whether NFPC mediates RGC axonal behaviour at other positions within the optic pathway remains unclear. Here we report that NFPC plays an important role in RGC axonogenesis, but not in intraretinal guidance. Moreover, axons with reduced NFPC levels exhibit insensitivity to Netrin-1, an attractive guidance cue expressed at the optic nerve head. Netrin-1 induces rapid turnover of NFPC localized to RGC growth cones, suggesting that the regulation of NFPC protein levels may underlie Netrin-1-mediated entry of RGC axons into the optic nerve head. At the tectum, we further reveal a function for NFPC in controlling RGC axonal entry into the final target area. Collectively, our results expand our understanding of the role of NFPC in RGC guidance and illustrate that this adhesion molecule contributes to axon behaviour at multiple points in the optic pathway.

## Introduction

During nervous system development, neurons extend axons that often navigate long distances to make contact with their synaptic targets. For instance, retinal ganglion cells (RGCs) synapse with neurons within the optic tectum [1]. To do this they must extend an axon out of the eye via the optic nerve, across the midline at the optic chiasm and along the optic tract to the tectum of the midbrain [2]. The navigation of RGC axons through this complex pathway is mediated by how growth cones interpret both cell-extrinsic guidance cues expressed at specific locations within the retinotectal pathway and by cell-intrinsic factors such as internal guidance receptors [3].

Cell adhesion molecules, such as members of the cadherin superfamily, can act as both intrinsic and extrinsic factors and also play a critical role with relation to promoting neuronal connectivity during development [4]. N-cadherin, for instance, has been implicated in processes including axon outgrowth [5], fasciculation [6], growth cone guidance [7], synaptogenesis [8] and dendrite arborization [6]. The protocadherins, a subgroup of the cadherin superfamily, have recently emerged as another set of factors important for the regulation of neural development [4]. Protocadherins have been shown to influence axon tract formation [9], axon target selection [10] and synaptic development [11]. With regards to the development of the retinotectal pathway, NFPC function has been shown to be critical for aspects of RGC axonal connectivity. This protein, which consists of seven cadherin-like ectodomains, a single transmembrane domain and a C-terminal intracellular domain [12], is expressed on RGCs within the retina, on RGC axons and by cells within the optic tract and tectum [13]. Moreover, functional studies have revealed that NFPC plays an important role in RGC axon initiation and elongation, as well as in mediating Sema3A-induced guidance of RGC axons within the mid-optic tract [13,14].

While performing the previous studies on NFPC, we noted expression of NFPC on RGC axons and within the tectum, indicating that this cell adhesion molecule may mediate additional aspects of retinotectal pathway development that have previously been unrecognized. Here we report that, although NFPC function does not appear to be necessary for intraretinal guidance of RGC axons, it does play a role in mediating chemotropic guidance to the attractive guidance cue Netrin-1, which is expressed at the optic nerve head. Netrin-1 induces rapid turnover of NFPC at the growth cone, indicative of changes in adhesion underlying guidance cue-mediated entry into the optic nerve head. At the entry point to the tectum, perturbations to NFPC expression, or inhibition of NFPC function with exogenously applied NFPC ectodomains, culminates in axons growing along the borders of the tectum, or looping aberrantly within the tectum. Together, these results show that NFPC is an important regulator of RGC axonal behaviour at multiple points within the retinotectal pathway.

## Materials and Methods

### *Xenopus* embryos

*Xenopus laevis* embryos were acquired through *in vitro* fertilization as described previously [14], and staged according to the normal tables of *Xenopus laevis* [15]. All animal experiments were approved by the Ethical Review Committee of the University of Cambridge and complied with Home Office guidelines.

### Lipofection

Stage 19 embryos were washed with 1x modified Barth’s saline (MBS) and aligned on freshly made agarose grids, before NFPC constructs were co-lipofected with a membrane-tethered GFP construct (GAP-GFP) into the eye field using previously described methods [16]. Briefly, DNA constructs were first mixed with the cationic lipid DOTAP at a 1:3 ratio (m/v), loaded into a glass micropipette and injected to the presumptive left hand eye field.

### Antisense morpholino oligonucleotides

Morpholino oligonucleotides were designed and synthesized by GeneTools. Morpholinos directed against the *Xenopus nfpc* start site (bold; NFPC-MO) were conjugated to FITC (3’ end) to make them detectable either directly or through immunostaining with an anti-FITC antibody. A standard non-specific sequence acted as a control morpholino (Con-MO). Control morpholinos were also conjugated to FITC. The efficacy of the NFPC-MO has previously been validated in retinal tissue [14].

Con-MO: 5’-CCTCTTACCTCAGTTACAATTTATA-3’

(125 μM for eye electroporation and 0.5 μM for tectum electroporation).

NFPC-MO: 5’-TCTGTGTCCCCTCAGTCCT**CAT**CAT-3’

(125 μM for eye electroporation, 0.5 μM for tectum electroporation).

### Electroporation

Electroporation of *Xenopus* embryos was performed as recently described with minor modifications [17]. Briefly, stage 22 embryos were anaesthetized with tricaine and transferred into modified Sylgard transfection chambers before 5–15 nl of diluted cDNA, plasmids, or antisense morpholino oligonucleotides were injected into the target area using a picospritzer. Immediately following this, electric pulses (8 pulses, 18 V, 50 ms pulse width with 1000 ms gap between pulses) were generated by a TSS20 OVODYNE electroporator. For the growth cone turning assay, embryos were allowed to recover at room temperature in 0.1xMBS for 1–2 h following electroporation of the retina, after which retinal primordia were dissected for culture. For electroporation of the tectum assay, morpholinos were injected directly into the tectum ventricle at stage 32 and electroporated toward the tectum neuropil. Embryos were then allowed to develop until stage 40, after which retinal axons were labelled with DiI loading.

### Fixation

*Xenopus* embryos were fixed in a 4% paraformaldehyde solution in phosphate buffered saline (PBS) for at least 3 h at room temperature or overnight at 4°C.

### Immunocytochemistry and intraretinal labelling

**Fig 1:**
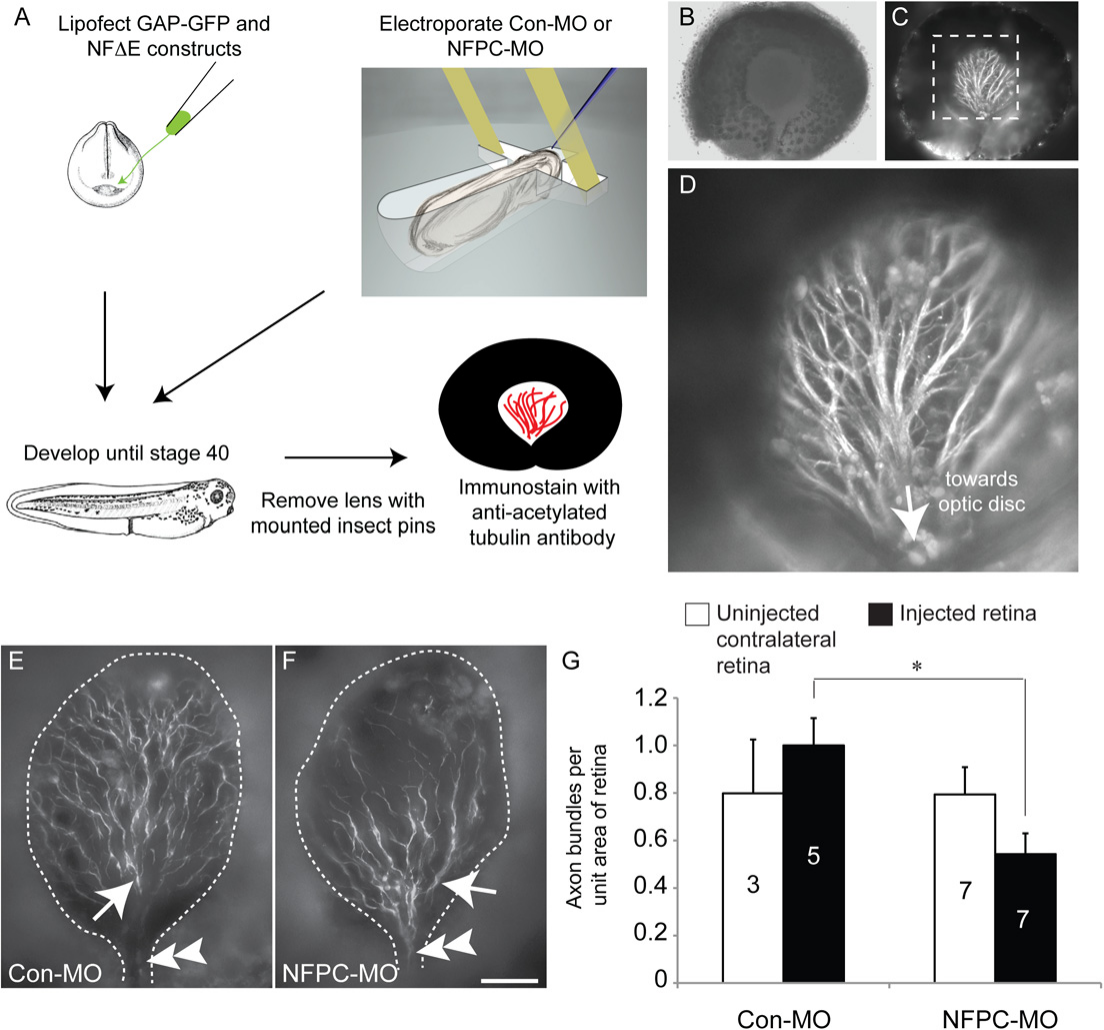
Electroporation of NFPC morpholinos disrupts RGC axon outgrowth *in vivo*. (**A**-**D**) A method for investigating intraretinal guidance. (**A**) RGCs were lipofected with either GAP-GFP or NFΔE constructs at stage 19, or electroporated with control (Con-MO) or NFPC morpholinos (NFPC-MO) at stage 24. Embryos were then allowed to develop until stage 40, at which time eyes were dissected from the embryo. After removal of the lens, immunostaining for acetylated α-tubulin was performed to mark retinal axons. (**B**) Brightfield image of an uninjected eye with lens removed. (**C**) Fluorescence labelling of the same eye as in **B**, revealing expression of acetylated α-tubulin in retinal axons. (**D**) Magnified view of the boxed region in **C**, showing immunolabelled retinal axons coursing towards the optic disc. (**E, F**) Acetylated α-tubulin staining in a retina loaded with the Con-MO (**E**) and a retina loaded with the NFPC-MO (**F**). In all cases analyzed regions of interest are delineated by dashed lines. Immunostaining reveals that in all cases RGC axon bundles (arrows in **E** and **F**) form and are oriented towards the optic disc (double arrowheads in **E** and **F**). However, quantification of the number of RGC axon bundles per unit area of the retina (**G**) reveals significantly reduced number of axon bundles in NFPC-MO-loaded retinae in comparison to Con-MO-loaded eyes. Values were normalized against the Con-MO group and the number of retinae analysed are presented within the bars. * *p* <0.05, Kruskal-Wallis test. Scale bar in **F**: 75 μm (**B, C**), 30 μm (**D**), 40 μm (**E, F**).

Standard immunostaining protocols were used with modifications for intraretinal antibody labelling [18]. Briefly, RGC axons were labelled intraretinally with an anti-acetylated α-tubulin antibody (1:400; mouse monoclonal, Sigma). For the antibody to access the retinal layers, lenses were first removed from freshly fixed embryos prior to immunostaining. Samples were then washed with PBS before being incubated with a Cy3-conjugated goat anti-mouse IgG secondary antibody (1:700, Sigma). For some samples, a small incision was made in the optic fissure so that the eye could be flat mounted to display more of the retinal surface. The quantification of the number of axon bundles was performed by counting clearly identifiable axon bundles within a manually selected region of interest (ROI; the visible retina after removing the lens), and dividing this by the area of the ROI (shown as dashed lines in our Fig 1 examples). Data were normalized to the Con-MO treated group. Immunocytochemical labelling of cultured retinal neurites was performed as described previously [19]. The primary antibody used was a rabbit polyclonal anti-NFPC antibody (1:500; gift from Assoc. Prof. Roger Bradley, Montana State University), and the secondary antibody used was an Alexa 488-conjugated goat anti-rabbit IgG (1:500; Molecular Probes).

### Quantitative immunofluorescence

Eye primordia were dissected from stage 24 embryos and cultured at 20°C for 24 h on coverslips coated with 10 μg/ml poly-L-lysine (Sigma) and 10 μg/ml laminin (Sigma). Immediately prior to the addition of Netrin-1 (300 ng/ml; a gift from Prof. Marc Tessier-Lavigne, The Rockefeller University), or vehicle control, the following pharmacological reagents were bath-applied to retinal cultures: 10 μg/ml α-amanatin (Calbiochem), 40 μM anisomycin (Sigma), 10 μM lactacystin (Sigma), 50 μM N-acetyl-leu-leu-norleu-al (LLnL, Sigma), 50 μM phenylarsine oxide (Sigma) and 100 nM monodansylcadaverine (Sigma). Immunostained cultures were assayed for fluorescence intensity with a 100x objective (Nikon) on a Nikon eclipse TE2000-U inverted microscope as previously described [19]. Briefly, a minimum of 30 non-collapsed growth cones were randomly picked, and examined for each sample group. Phase and fluorescence images were captured using a Hamamatsu camera. Outlines of growth cones (phase) were traced digitally in Openlab (Improvision) and used to calculate the mean fluorescence (pixel) intensity per unit area in the fluorescent image of the outlined growth cone and the background. To give final intensity measurements, subtractions of background from the growth cone values were done using Excel (Microsoft). Values are presented as mean ± standard error of the mean from a minimum of 4 independent experiments and are normalized to vehicle-stimulated means. Significance was determined by the Kruskal-Wallis test for non-parametric variance.

### Growth cone turning assay

*In vitro* growth cone turning assays were performed as previously described [20,21]. Briefly, a 1000-fold gradient (with a radius of 100 μm) of Netrin-1 diluted in culture medium (CM; this was also used for the vehicle-only control) was generated by pulsatile ejection out of a micropipette (pulled to a final tip diameter of 1 μm) using a picospritzer (General Valve). Using a 20x objective, growth cones from 24 h retinal cultures were positioned at a distance of 100 μm from the micropipette tip at an angle of 45° relative to the initial direction of the axon shaft. RGC growth cones were examined at 100x using a fluorescence microscope prior to the assay to verify the presence of the morpholinos. Selected growth cones were exposed to a gradient of Netrin-1 (or culture medium) for 1 h, and images were captured every 10 min. The turning angles of growth cones that displayed a minimum net extension of 10 μm were measured using Openlab or Image J software (NIH) as described previously [20]. The final patterns of extension were traced to create trajectory plots. A comparison of means was determined by the Mann-Whitney U statistic and the Kolmogorov-Smirnov test was used to compare the normality of distributions.

### Open brain incubation with NFPC ectodomains

To inhibit the homophilic interaction of axon and tectum expressed NFPC, the dominant negative NFPC ectodomain (NFPC-Fc) protein and control-Fc protein were produced and harvested as described previously [14]. Open brain experiments were performed on stage 35 embryos as previously described [22]. Briefly, the skin and dura were removed from above the target area to be exposed with mounted insect pins. Embryos were then transferred to a 4-well dish containing 500 μl of 1.3x MBS and MS222 containing control-Fc or NFPC-Fc (1:100). Embryos were incubated until stage 40 prior to fixation and DiI axon labelling.

### DiI filling

The lipophilic dye, DiI, was used to label the entire population of retinal axons such that the path followed by the RGC axons could be visualized in both control and test conditions. 25 mg/μl (m/v) of DiI crystals dissolved in ethanol was loaded into a micropipette, and injected into the eye as described previously [14]. After dye transfer, samples were immediately visualized with an upright fluorescence or confocal microscope.

### Statistical analysis

A minimum of three independent trials were conducted for each experiment. Data were analysed with either the Kolmogorov-Smirnov, Kruskal-Wallis or Fisher’s exact test as appropriate. Error bars represent the standard error of the mean.

## Results

### Downregulation of NFPC does not disrupt intraretinal guidance of RGC axons

NFPC is expressed within the developing retina by RGCs [13,14]. We have previously shown, using a dominant-negative NFPC construct (NFΔE), that both axon and dendrite formation are reduced when NFPC function is impaired [13]. However, whether inhibition of NFPC function perturbs axon navigation across the retinal surface to the optic nerve head (the future optic disc), known as intraretinal axon guidance, is unknown. To address this we adopted a method for investigating the navigation of RGC axons towards the optic disc (Fig 1A–1D) [18]. NFPC function was knocked-down within RGCs by either lipofection of the dominant negative NFΔE construct into the optic primordium at stage 19, or by electroporating an anti-NFPC morpholino (NFPC-MO) into the retina at stage 24. The efficacy of the NFPC-MO in retinal tissue has previously been demonstrated [14]. Embryos were then grown until stage 40, whereupon the lens was removed from the treated eye and retinal axons were labelled in wholemount retinae by immunostaining for acetylated α-tubulin [23]. The growth of RGC axons in uninjected retinae (data not shown), as well as those treated with the control morpholinos (Con-MO; Fig 1E and 1G) or GAP-GFP alone (S1 Fig), was clearly oriented towards the optic disc prior to their entry into the optic nerve head. However, analysis of the number of RGC axon bundles in those retinae treated with the NFPC-MO revealed significantly reduced axon bundle numbers, consistent with previous reports detailing deficits in RGC axonogenesis in retinae with perturbed NFPC function ([13] Fig 1F and 1G). Similar results were obtained following lipofection of the NFΔE construct into stage 19 retinae (S2 Fig). Interestingly, however, there were no obvious deficits in RGC intraretinal guidance, as those axon bundles evident in treated retinae were appropriately oriented towards the optic disc (Fig 1F; S2 Fig), indicating that NFPC may not modulate this aspect of RGC axon guidance.

### Knockdown of NFPC abolishes retinal neurite sensitivity to Netrin-1

We have previously reported that the majority of RGCs with perturbed NFPC function prior to axon extension fail to extend an axon beyond the retina [13]. The above results show that this defect is not because the affected axons are misguided in the retina, as they appear to grow directly towards the optic disc. Thus, we conclude that the failure of these axons to exit the eye happens at the point where they join the optic nerve. To investigate whether NFPC contributes to retinal axon behaviour in response to chemotropic guidance cues expressed at this point in the optic pathway, we examined whether RGC axons with compromised NFPC function show altered chemotropic responses to Netrin-1, which is specifically expressed at the optic nerve head, and which is pivotal for promoting RGC axonal exit from the eye [24,25].

It has been reported that RGC neurites cultured from young embryos navigate towards a point source of Netrin-1 [23,24]. We first repeated these findings to ensure that our source of Netrin-1 was indeed chemoattractive to retinal growth cones. In line with previous findings [18,23,24], stage 22 retinal growth cones cultured for 24 h exhibited chemoattractive turning towards a Netrin-1 gradient, whereas retinal neurites displayed no directional bias towards a gradient of vehicle solution (S3 Fig). To investigate the contribution of NFPC to Netrin-1-mediated guidance within the turning assay, we used electroporation of the anti-NFPC-MO oligonucleotides conjugated to FITC, which enabled the identification of neurons/neurites containing the morpholino (S4 Fig). NFΔE-expressing cells, on the other hand, can only be identified *post hoc* through immunostaining post-fixation. As a control, we first investigated whether electroporation itself could affect the growth cone turning response, as there is extensive evidence implicating membrane potential and voltage-gated channels in influencing turning [26–28]. Neurites from mock-electroporated retinae exhibited net turning towards the source of Netrin-1 (S3 Fig), illustrating that electroporation *per se* did not influence turning behaviour within the assay.

**Fig 2:**
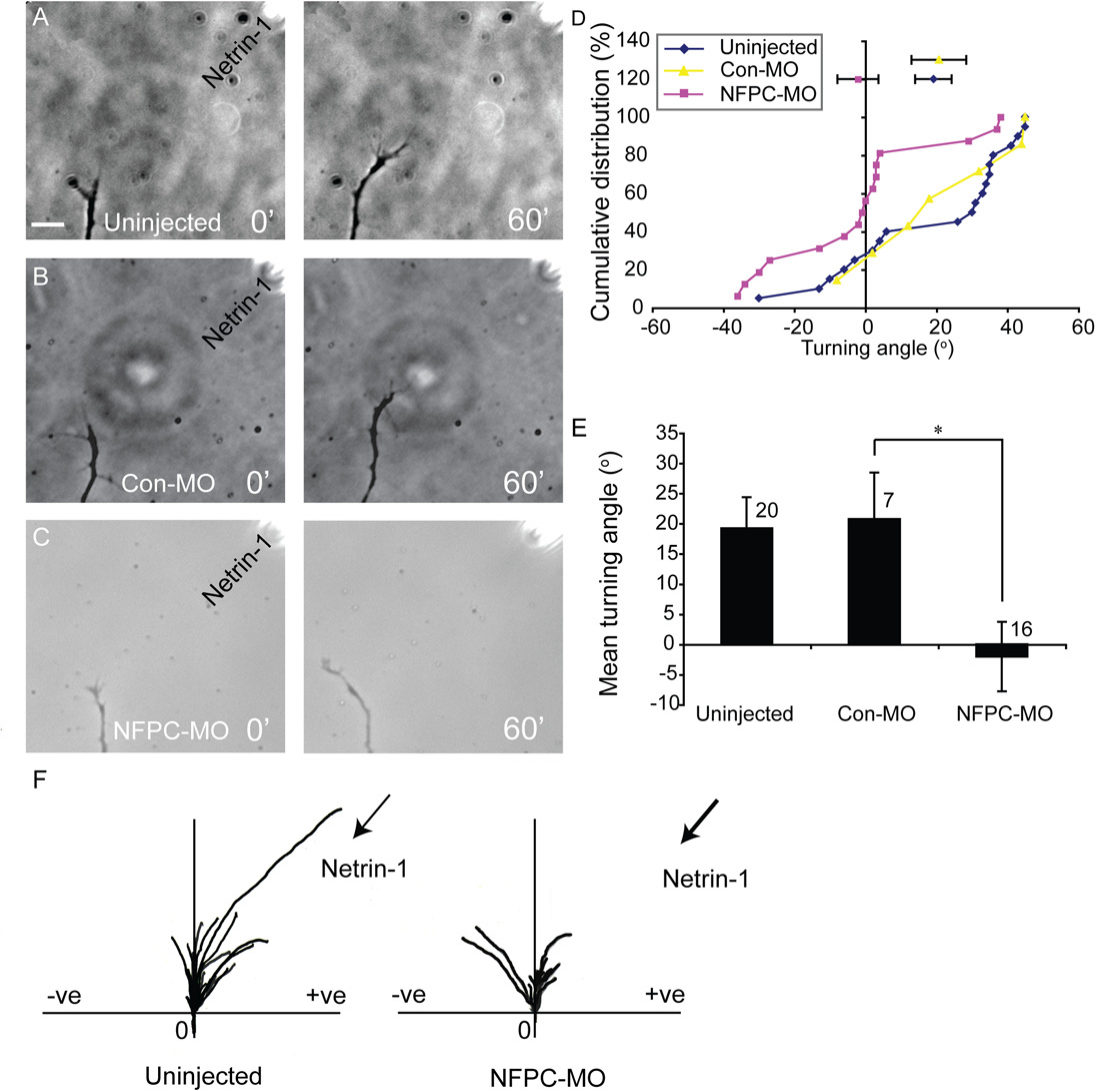
Cultured retinal growth cones loaded with NFPC morpholino do not exhibit chemoattraction to Netrin-1. (**A-C**) Phase contrast images of retinal neurites cultured from uninjected (**A**), control morpholino-loaded (Con-MO; **B**) and NFPC morpholino-loaded (NFPC-MO; **C**) retinae. Neurites from uninjected (**A**) and Con-MO-loaded (**B**) retinae exhibit robust turning towards the point source of Netrin-1. Neurites loaded with the NFPC-MO, however, are not attracted towards Netrin-1 (**C**). (**D**) Cumulative distributions of turning angles of each sample group. (**E**) Mean turning angles of the experimental groups reveals that, whereas uninjected and Con-MO-loaded neurites exhibit attraction to Netrin-1, NFPC-MO-loaded neurites do not. * *p* < 0.05, Kolmogorov-Smirnov test. Panel **F** shows a summary of the trajectory plots from the uninjected and NFPC-MO experimental groups exposed to Netrin-1. Each line represents a single growth cone trajectory; the origin represents the centre of the growth cone at 0 min, and positive (+ve) and negative (-ve) turning angles are indicated. Scale bar in **A**: 10 μm.

To assay for the role of NFPC in Netrin-1-mediated growth cone turning, we selected fluorescently labelled Con-MO-containing (FITC-tagged Con-MO) or NFPC-MO-containing (FITC-tagged) neurites (S4 Fig). Whereas neurites from uninjected retinae or those electroporated with the Con-MO demonstrated chemoattraction to Netrin-1 in the turning assay, retinal neurites loaded with the NFPC-MO exhibited no turning bias (Fig 2). Netrin-1 is known to promote both elongation and turning *in vitro* [24]. Therefore, we quantified the average rate of neurite elongation between the Con-MO-loaded group (24.3 μm/hr; 7 neurites) and the NFPC-MO-loaded group (23.3 μm/hr; 13 neurites). There was no significant difference in average neurite elongation between the groups (*p* > 0.05, Kolmogorov-Smirnov test), suggesting that the failure of NFPC-MO-loaded neurites to turn towards a gradient of Netrin-1 was not due to a non-specific defect in elongation. Moreover, we have previously measured the extension rate of NFPC-deficient axons growing through the optic tract using live imaging and found that they extend at the same rate as controls in the ventral optic tract [14]. Collectively, the findings in the *in vitro* turning assay, coupled with the failure of the majority of NFΔE-expressing RGC axons to exit the retina [13], points to a role for NFPC in the Netrin-1-mediated entry of retinal axons into the optic nerve head.

### Netrin-1 dynamically regulates NFPC in cultured retinal growth cones

**Fig 3:**
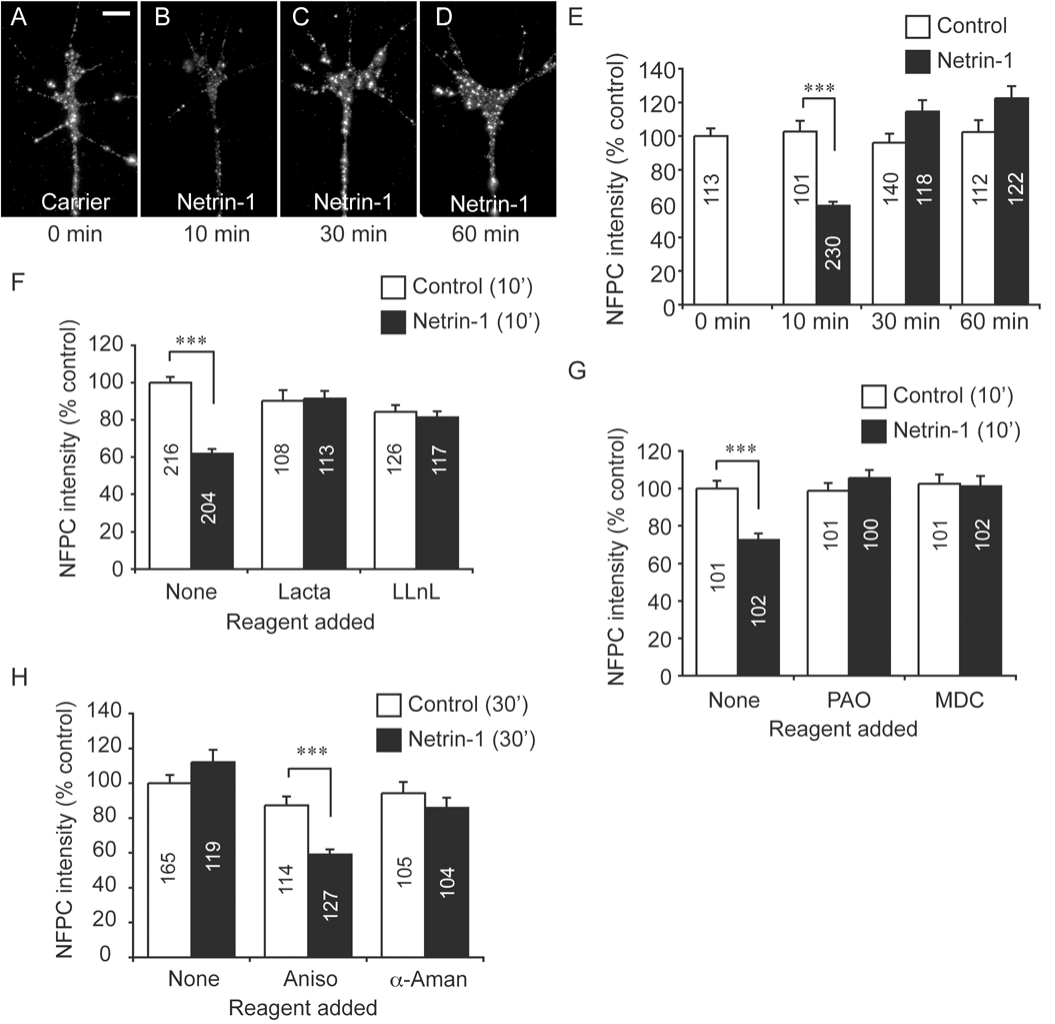
Netrin-1 dynamically regulates NFPC in retinal growth cones. (**A-D**) Cultured stage 24 retinal neurites were stimulated with Netrin-1 or a vector-only control for 0 (**A**), 10 (**B**), 30 (**C**) or 60 (**D**) min, and then assayed for NFPC expression via immunofluorescence labelling. Panels **E**-**H** reveal the quantification of immunofluorescence as an indicator of total NFPC levels within the growth cone (open bars–vector only control; black bars–Netrin-1 treatment). In each case, data were normalized to the 0 min control treatment. (**E**) Netrin-1 induced a significant decrease in the levels of NFPC within the growth cone after 10 min. By 30 min, however, NFPC localized to the growth cone had returned to levels comparable to that in the control. (**F**) The decrease in NFPC immunoreactivity localized to the growth cone after 10 min was abolished when explants were pre-treated with the proteasomal inhibitors lactacystin (Lacta) or LLnL. (**G**) The decrease in NFPC localized to the growth cone after 10 min was also abolished when the explants were treated with the endocytosis inhibitors phenylarsine oxide (PAO) or monodansylcadaverine (MDC). (**H**) Blocking protein translation with anisomycin (Aniso) in isolated retinal neurites suppressed the recovery in growth cone NFPC levels seen after 30 min of Netrin-1 exposure. However, inhibition of transcription with α-amanatin (α-aman) did not prevent the recovery of growth cone NFPC levels. *** *p* < 0.001, Kruskal Wallis test. Numbers within the bars indicate the number of growth cones assayed. Scale bar in **A**: 5 μm.

Mechanistically, RGC growth cone responses to Netrin-1 have been shown to require protein turnover involving both local protein translation and degradation [29–31]. For example, attractive guidance towards Netrin-1 or BDNF requires local translation of *β-actin* mRNA [31,32]. Given the expression of *nfpc* mRNA within RGC axons in the optic fibre layer of the retina [13], and our finding that blocking retinal neurites with NFPC-MO abolishes Netrin-1-induced chemoattraction, we sought to determine whether Netrin-1 application to retinal neurites elicited changes to the level of NFPC localized to the growth cone. To do this we analysed growth cones from stage 24 retinae (prior to axon initiation *in vivo*) that had been cultured for 24 h on a laminin substrate, as laminin is expressed strongly in the optic fibre layer [23]. Cultured growth cones were stimulated with bath-applied Netrin-1 for times ranging between 10 and 60 min. Quantitative immunofluorescence was then used to determine the total level of NFPC localized to the growth cone. When normalized to vehicle control levels, Netrin-1 induced a rapid and highly significant decrease in NFPC after 10 min, but by 30 min the level of NFPC had returned to that seen prior to stimulation (Fig 3A–3E). This suggests that Netrin-1 induces a rapid turnover of this adhesion molecule. The reduction in NFPC levels in response to Netrin-1 likely occurs via protein degradation, as this process has previously been shown to be required for rapid chemotropic responses to Netrin-1 [29]. Indeed, the proteasomal inhibitors lactacystin and LLnL both abolished the decrease in NFPC levels observed after 10 min, implicating protein degradation in the decrease in NFPC levels induced by Netrin-1 (Fig 3F).

As NFPC is mainly expressed on the membranes of growth cones and is degraded in response to Netrin-1, this suggests NFPC is first internalised before it is targeted to the proteasome. To test this, we pre-incubated growth cones with either the broad endocytosis inhibitor phenylarsine oxide or the clathrin-mediated endocytosis inhibitor monodansylcadaverine. Both treatments effectively blocked the reduction in NFPC immunoreactivity seen after 10 min of stimulation with Netrin-1 (Fig 3G). These findings suggest that endocytosis of surface NFPC and degradation of this cell adhesion molecule are both essential components of the growth cone response to Netrin-1.

We also observed that, after 30 min of netrin-1 application, the level of NFPC protein localized to the growth cone had returned to a range equivalent to that observed prior to stimulation (Fig 3E). This recovery in NFPC levels could arise from either transport of NFPC from the soma, or by translation of *nfpc* mRNA, which is abundant in RGC axons and growth cones. To differentiate between these possibilities, we first pre-incubated our samples with either α-amanatin, a transcriptional inhibitor, or anisomycin, a translational inhibitor, and then stimulated retinal growth cones with Netrin-1 for 30 min. Only anisomycin inhibited the recovery in NFPC immunoreactivity observed after Netrin-1 stimulation, suggesting that the translation of NFPC underlies the recovery in protein levels (data not shown). Furthermore, when we repeated this experiment on retinal neurites that had been separated from their cell soma by transection prior to the assay, we saw a similar effect (Fig 3H), providing evidence that it is *local* translation of *nfpc* mRNA within the growth cone that underlies the recovery in NFPC protein levels following Netrin-1 stimulation. This is consistent with reported findings that NFPC is synthesized locally in growth cones *in vivo* [14]. Taken together, these findings suggest that Netrin-1 dynamically regulates the level of NFPC protein localized to the growth cone through local degradation and synthesis, and that this could contribute to Netrin-1-mediated entry of retinal axons into the optic nerve head.

### NFPC regulates axon entry into the optic tectum

**Fig 4:**
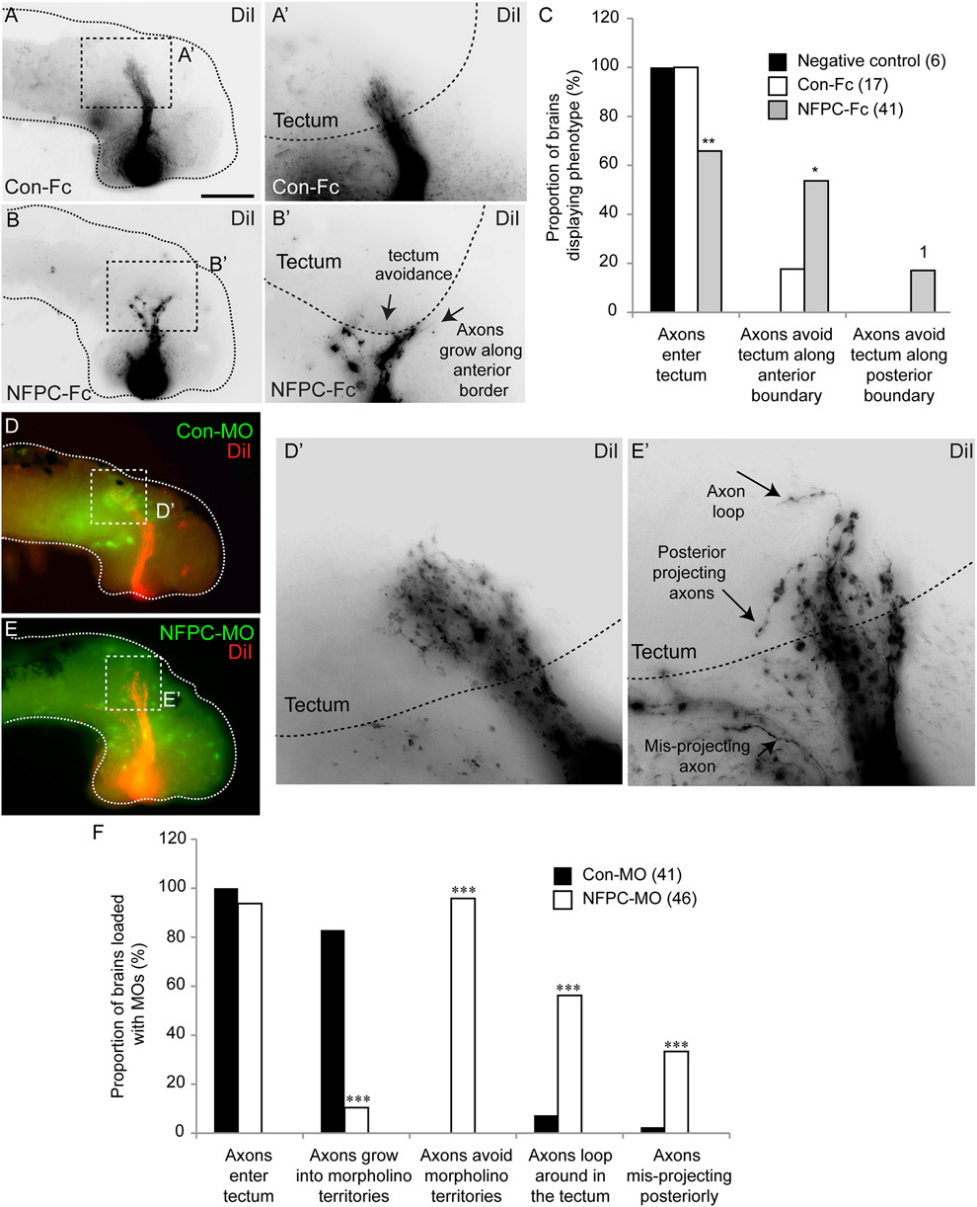
Perturbation of NFPC binding leads to tectum entry defects. Open brain embryos were incubated with Con-Fc (A) or NFPC-Fc (B) from stage 35. At stage 40, retinal axons were labelled with DiI. Brains were then dissected and mounted in the contralateral view to enable visualization of the optic tract. Inverse greyscale images show that the axon bundle trajectories of Con-Fc-treated brains appeared normal (**A**, higher magnification shown in **A’**), with RGC axons entering the tectum normally (the tectum is delineated with a dashed line in **A’**). Brains treated with the NFPC-Fc ectodomain construct (**B**), however, exhibit various phenotypes including axons avoiding the tectum and growing along the anterior tectal boundary (**B’**). (**C**) Graph showing the proportion of brains incubated with the NFPC-Fc peptide that display axons avoiding either the anterior or posterior boundary of the tectum. Statistical significance was calculated against the Con-Fc proportions. * *p*<0.05, ** *p*<0.01, ^1^p = 0.0934, Fisher’s exact test (6 independent experiments). Brains electroporated with the Con-MO within the optic tectum (**D**) do not exhibit defects in axon pathfinding, as RGC axons grew through the electroporated region into the tectum (**D’** is an inverse greyscale image showing the trajectory of DiI-filled RGC axons). Panel **E** shows a representative image of a brain electroporated with the NFPC-MO within the tectum. Perturbation of NFPC binding culminated in phenotypes including looping and projection along the posterior tectal boundary (**E’** is an inverse greyscale image showing the trajectory of DiI-filled RGC axons). Panel **F** reveals the proportion of brains loaded with the NFPC-MO that exhibit abnormal projections into the tectum. Statistical significance was calculated against Con-MO proportions. *** *p*<0.001, Fisher’s exact test (7 independent experiments). Scale bar in **A**: 300 μm (**A**, **B**, **D**, **E**), 75 μm (**A’**, **B’**), 50 μm (**D’**, **E’**).

As well as being strongly expressed within the eye, NFPC is also present within the dorsal optic tract and the optic tectum [14], suggestive of additional roles for this cell adhesion molecule beyond regulating axonogenesis and axonal exit from the retina. Indeed, a recent study has shown that NFPC function is critical for the navigation of retinal axons in the mid-optic tract. For instance, inhibiting NFPC’s homophilic interactions with a blocking peptide containing the NFPC ectodomain fused to a Fc fragment (NFPC-Fc) in open brain preparations culminated in retinal axon pathfinding defects in the caudal turn portion of the mid-optic tract [14]. In this study we also noted that some axons skirted around the border of the tectum rather than entering it, indicative of tectal avoidance. To expand on these preliminary findings, we exposed the brains of stage 35 embryos to either the control-Fc, or NFPC-Fc, and analyzed axonal behavior at stage 40. Brains treated with Con-Fc exhibited normal growth of retinal axons into the tectum (Fig 4A and 4C). However, embryos treated with the NFPC-Fc ectodomain construct displayed defects in guidance, including avoidance of the tectal boundary, and failure of entry to the tectum (Fig 4B and 4C). Quantification of these defects revealed a significant proportion of axons making aberrant guidance decisions at the tectum when treated with the NFPC ectodomain (Fig 4C).

We have previously shown, using open brain preparations treated in this way, that RGC axons display guidance defects at the mid-optic tract [14]. It is therefore possible that the tectal entry deficits are simply a product of this earlier guidance defect. To address this, we took advantage of the fact that NFPC is expressed both on RGC axons, and within the tectum itself [13]. Homophilic interactions between NFPC-expressing RGC axons and the NFPC-expressing substrate within the mid-optic tract are critical for axon navigation within this portion of the retinotectal pathway [14]. As such, we postulated that manipulating the expression of the homophilic NFPC ligand within the tectum alone would provide an avenue to address the role of NFPC in RGC axon entry into this area without the potential confounds arising from earlier guidance deficits. We therefore electroporated the Con-MO or the NFPC-MO directly into the tectum at stage 32, prior to retinal axon entry into the lateral optic tract. At stage 40, retinal axons were labelled with DiI, and their projection into the tectum was assessed. Electroporation of the Con-MO did not affect the entry of DiI-labelled retinal axon bundles into the tectum (Fig 4D). However, the electroporation of the NFPC-MO culminated in a range of phenotypes (Fig 4E). Firstly, at a population level, retinal axons grew into regions containing the NFPC-MO far less frequently (10.4%, n = 48, *p* < 0.001, Fisher’s exact test; Fig 4F) than into Con-MO regions (82.9%, n = 41), suggesting that retinal axons were avoiding these regions. Moreover, we observed two other main phenotypes at an individual axon level: looping, where axons grew in a circular path within the tectum, and aberrant axonal growth towards the posterior tectal boundary. Axonal looping was observed at a significantly higher level in those embryos in which NFPC expression had been inhibited within the tectum when compared to controls (56.2% in NFPC-MO-treated samples; 7.3% of Con-MO-treated samples, *p* < 0.001, Fisher’s exact test, Fig 4F). Similarly, there was a significantly higher level of posterior growth in NFPC-MO-treated samples when compared to controls (*p* < 0.001, Fisher’s exact test, Fig 4F). This indicates that the NFPC-mediated interaction of retinal axons and the tectum substrate likely provides a signal for RGC axon invasion of target area for subsequent synaptic connectivity.

## Discussion

NFPC has been shown to mediate RGC axon initiation and elongation, and more recently it has been demonstrated that it is upregulated in response to the guidance cue Sema3A, thereby mediating axonal pathfinding in the mid-optic tract [13,14]. Here we extend these studies to reveal a role for NFPC at additional locations within the retinotectal pathway. Using a suite of *in vitro* and *in vivo* techniques, coupled with perturbation of NFPC function, we demonstrate that Netrin-1-induced attraction of RGC neurites is abolished upon reduction in growth cone NFPC levels, suggestive of a role for NFPC in mediating RGC axon entry to the optic nerve head. Reciprocally, Netrin-1 exposure leads to the rapid endocytosis and degradation of NFPC, which may help RGCs axons exit the retina once they have made to turn into the optic nerve head. Furthermore, we demonstrate that NFPC is required for the correct entry of RGC axons into the tectum. Collectively, this study, in association with previous reports, suggests a model whereby NFPC is required at key locations within the retinotectal pathway, including for RGC axonogenesis [13], for sensitivity towards Netrin-1 expressed at the optic nerve head (this study), for axon pathfinding at the mid-optic tract [14] and for RGC axonal entry into the tectum (this study).

After axonogenesis, nascent RGC axons become confined to the optic fibre layer and converge at the optic disc prior to exiting the eye via the optic nerve head. The process of intraretinal guidance of RGC axons towards the optic disc has been shown to be influenced by adhesion molecules such as L1 and NCAM [33], as well as by both positive signals, such as contact with the end feet of Müller glia [34,35] and existing retinal pioneering axons [36], and negative signals, including surround repulsion by chemotropic cues such as Slit1 and Slit2 [37] and by chondroitin sulphate proteoglycans [38]. Moreover, when axons reach the optic nerve head, they are directed to turn into this region by Netrin-1, which is specifically expressed at this choice point [18,25]. Our current findings reveal a role for this protocadherin in regulating RGC axon responses to Netrin-1, but not in intraretinal axon guidance, as RGC axons with perturbed NFPC function or NFPC protein levels appeared to navigate with fidelity towards the optic disc. Our previous findings analyzing RGC axons at an individual level revealed that, although some axons with impaired NFPC function failed to extend an axon, the majority did extend an axon towards the optic disc, but only a small a proportion of these axons exited the eye via the optic nerve head [13]. This suggests that the intraretinal guidance of axons does not require NFPC function. Instead, our present data reveal a role for NFPC in chemotropic responses to Netrin-1, an attractive guidance cue expressed specifically at the optic nerve head and implicated in mediating the exit of retinal axons from the eye [24,25]. Our data are consistent with the hypothesis that the failure of NFPC-deficient axons to exit the retina is due to reduced sensitivity to Netrin-1, although at this stage we cannot rule out the possibility that other, non-Netrin-1-mediated mechanisms contribute to this phenotype.

Our data also indicate that Netrin-1 induces turnover of NFPC localized to the growth cone. Previously we have used an *in vitro* collapse assay to demonstrate that Netrin-1 elicits ligand-specific desensitization of *Xenopus* retinal growth cones that is dependent on endocytosis, followed by resensitization that is dependent on local protein synthesis [39]. Our finding that Netrin-1 induces a decrease in growth cone NFPC that is endocytosis-dependent, followed by a return to basal levels that is protein synthesis-dependent, points to dynamic regulation of NFPC levels contributing to the response of the growth cone to Netrin-1. We speculate that the Netrin-1-induced drop in NFPC levels seen at 10 minutes *in vitro* causes a temporary loss of Netrin-1 sensitivity. This, coupled with the previously reported laminin-1-mediated inhibition of Netrin-1 [18], may switch off/down growth cone attraction to Netrin-1, enabling axons to move through the attractive intermediate target of the optic nerve head. The subsequent recovery in *de novo* synthesized NFPC may further play a role in facilitating the next phase of the growth cone’s journey. This regulation by Netrin-1 is also in contrast to rapid NFPC increases stimulated by another guidance cue, Sema3A, in the mid-optic tract [14], which offers potential evidence of the differential regulation of the same mRNA species by various cues along the pathway for specific functions required locally by the growth cone.

Indeed, Sema3A-induced local translation of NFPC was recently proposed to facilitate the caudal turn that RGC axons must make to navigate towards the tectum [14]. Using an NFPC blocking peptide (NFPC-Fc) in an open brain preparation, the previous study further suggested that NFPC expressed within the substrate of the optic tract was needed to enable turning of RGC growth cones at this point in the retinotectal pathway. Our findings extend these observations, indicating that blockade of NFPC function also inhibits RGC axon entry into the tectum, a process that is normally preceded by a significant drop in axonal growth rate, indicative of tectal entry representing a key choice-point for navigating axons [2]. Despite intensive study of retinotectal pathway development, surprisingly little is known about the molecular determinants underpinning the growth of axons into this region. The expression of Sema3A in the posterior and ventroposterior regions of the tectum has been postulated to channel RGC axons into the tectum [40], and ectopic application of FGF2 or heparin sulfates culminates in mistargeting of RGC axons [41,42]. The depletion of NFPC within the substrate of the tectum (NFPC morpholinos) and with the function-blocking peptide in open brain preparations indicate that NFPC is also required for axons to correctly navigate this critical choice point. Whether Sema3A-mediated changes in tectal NFPC interactions are required for axon targeting remains an intriguing question.

We observed two main phenotypes following inhibition of NFPC function within the tectum, namely misprojections along the border of the tectum, and looping of axons within the tectum. Whether these represent a phenotypic spectrum related to differing levels of NFPC inhibition between RGC axons and the substrate, or whether they are distinct phenotypes that represent deficiencies in different aspects of RGC behaviour at this choice point is unclear. However, these findings do suggest that the ability of retinal growth cones to make specific NFPC-mediated interactions is critical for crossing the tectal boundary and for cessation of growth within the tectum. Given the strong expression of NFPC within the tectum [13], it will be interesting to address whether NFPC also plays a role in other aspects of retinal pathway development, including topographic mapping, target selection and synaptogenesis. Indeed, previous work has shown that protocadherins are involved in processes including target selection [10], neuronal survival [43–45] and the development of synapses [11]. Experiments using knockdown of NFPC function in a temporally controlled fashion, coupled to live imaging techniques will, in the future, enable the contribution of NFPC function to these events to be investigated.

## Supporting Information

S1 Fig Lipofection of GAP-GFP does not disrupt RGC axon outgrowth *in vivo*. (**A**) An example of an uninjected eye. Immunostaining with acetylated α-tubulin (red) revealed a normal pattern of axon outgrowth (arrow) that converged on the optic disc (double arrowhead) to exit the eye. (**B**) Lipofection with a GAP-GFP construct did not affect the number or the directionality of axon bundles (arrow) converging on the optic disc (double arrowhead). Exposed retinae are marked by dashed white lines. Scale bar in **B**: 30 μm.
(TIF)

S2 Fig Dominant negative disruption of NFPC activity disrupts RGC axon outgrowth *in vivo*. (**A**) An example of a contralateral, uninjected eye. Immunostaining with acetylated α-tubulin (red) revealed a normal pattern of axon outgrowth (arrow) converging on the optic disc (double arrowhead) to exit the eye. (**B**) Lipofection with the NFΔE construct culminated in disrupted retinal axon outgrowth, with these retinae exhibiting a significantly reduced number of axon bundles per unit area of retina in comparison with the GAP-GFP-expressing controls (**C**; * *p* < 0.05, Kruskal-Wallis test. Values were normalized against the GAP-GFP-injected group and the number of retinae analysed are presented within the bars.). However, the remaining axon bundles (arrows in **B**) converged on the optic disc (double arrowhead). Scale bar in **B**: 30 μm.
(TIF)

S3 Fig Cultured retinal neurites exhibit chemoattraction to Netrin-1. (**A-C**) Phase contrast images of retinal neurites from uninjected (**A, B**) and mock electroporated (**C**) retinae exposed to either a culture medium control (CM; **A**) or netrin-1 (**B, C**). (**D**) Cumulative distributions of turning angles of each sample group. (**E**) Mean turning angles of the experimental groups reveals that, whereas uninjected neurites exposed to CM did not exhibit any turning bias, neurites exposed to Netrin-1 were attracted to this guidance cue. * *p* < 0.05, Kolmogorov-Smirnov test. Scale bar in **A**: 10 μm.
(TIF)

S4 Fig Protocol for preparing morpholino-loaded RGC growth cones. (**A**) Embryonic retinal primordia were electroporated with either a control, FITC-tagged morpholino (Con-MO) or a FITC-tagged anti-NFPC morpholino (NFPC-MO) using a specifically designed electroporation chamber. Eyes were then removed and cultured *in vitro* for 24 h. Examples of Con-MO-loaded (**B**, red) and NFPC-MO-loaded (**C**, green) neurites are shown.
(TIF)
