# Supplementary figures and images for: NF-Protocadherin Regulates Retinal Ganglion Cell Axon Behaviour in the Developing Visual System
Source: PLoS One. 2015 Oct 21;10(10):e0141290. doi: 10.1371/journal.pone.0141290 (PMC4619323; doi:10.1371/journal.pone.0141290)

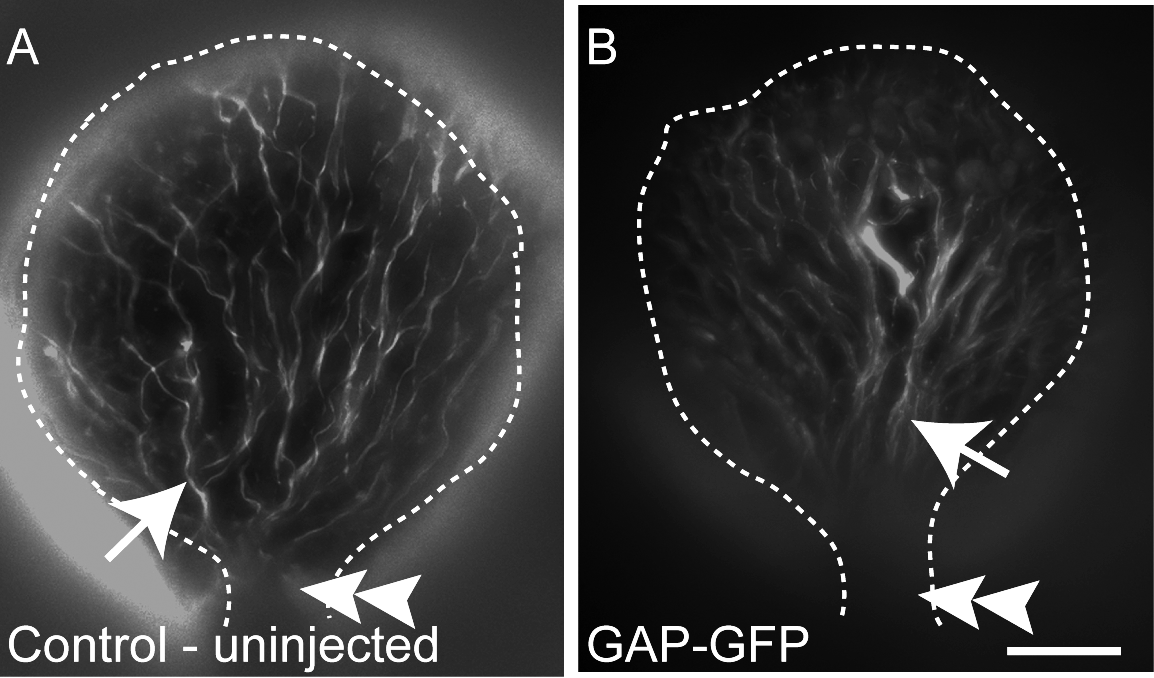

Supplement: S1 Fig — (A) An example of an uninjected eye. Immunostaining with acetylated α-tubulin (red) revealed a normal pattern of axon outgrowth (arrow) that converged on the optic disc (double arrowhead) to exit the eye. (B) Lipofection with a GAP-GFP construct did not affect the number or the directionality of axon bundles (arrow) converging on the optic disc (double arrowhead). Exposed retinae are marked by dashed white lines. Scale bar in B: 30 μm. (TIF) [file pone.0141290.s001.tif]

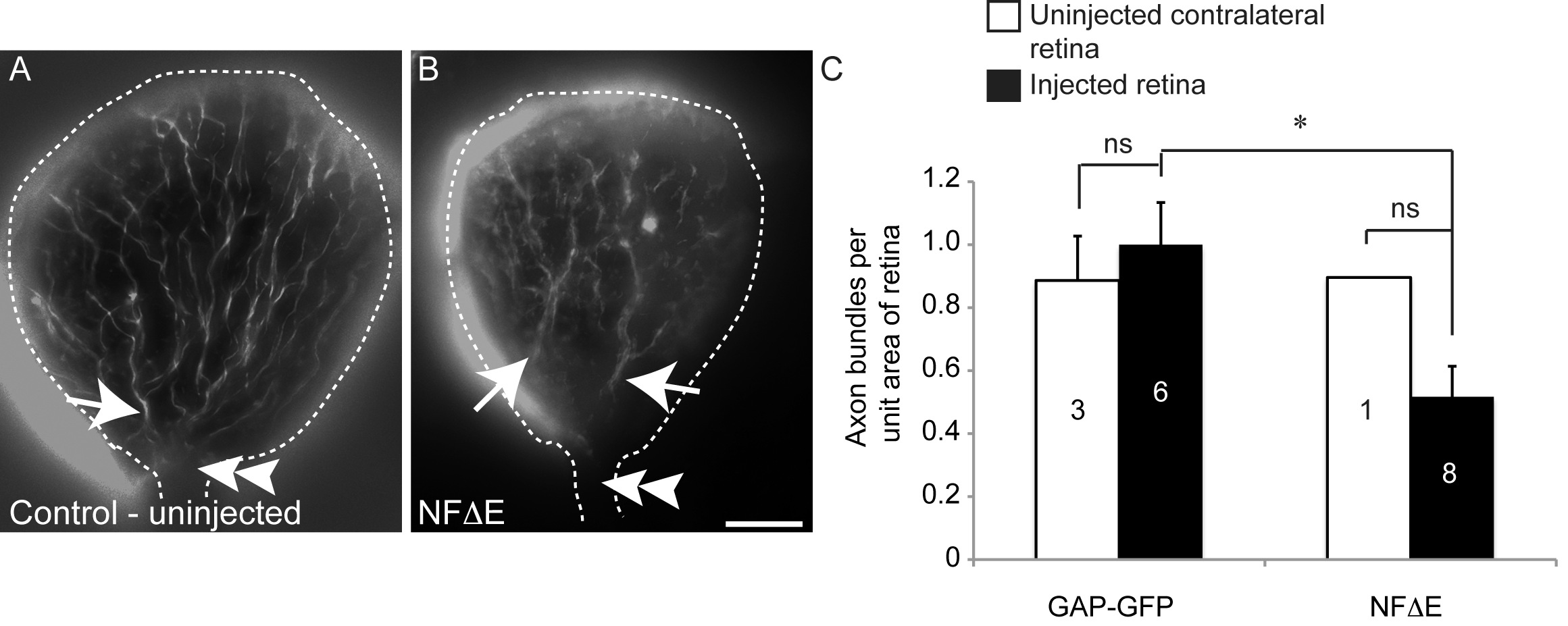

Supplement: S2 Fig — (A) An example of a contralateral, uninjected eye. Immunostaining with acetylated α-tubulin (red) revealed a normal pattern of axon outgrowth (arrow) converging on the optic disc (double arrowhead) to exit the eye. (B) Lipofection with the NFΔE construct culminated in disrupted retinal axon outgrowth, with these retinae exhibiting a significantly reduced number of axon bundles per unit area of retina in comparison with the GAP-GFP-expressing controls (C; * p < 0.05, Kruskal-Wallis test. Values were normalized against the GAP-GFP-injected group and the number of retinae analysed are presented within the bars.). However, the remaining axon bundles (arrows in B) converged on the optic disc (double arrowhead). Scale bar in B: 30 μm. (TIF) [file pone.0141290.s002.tif]

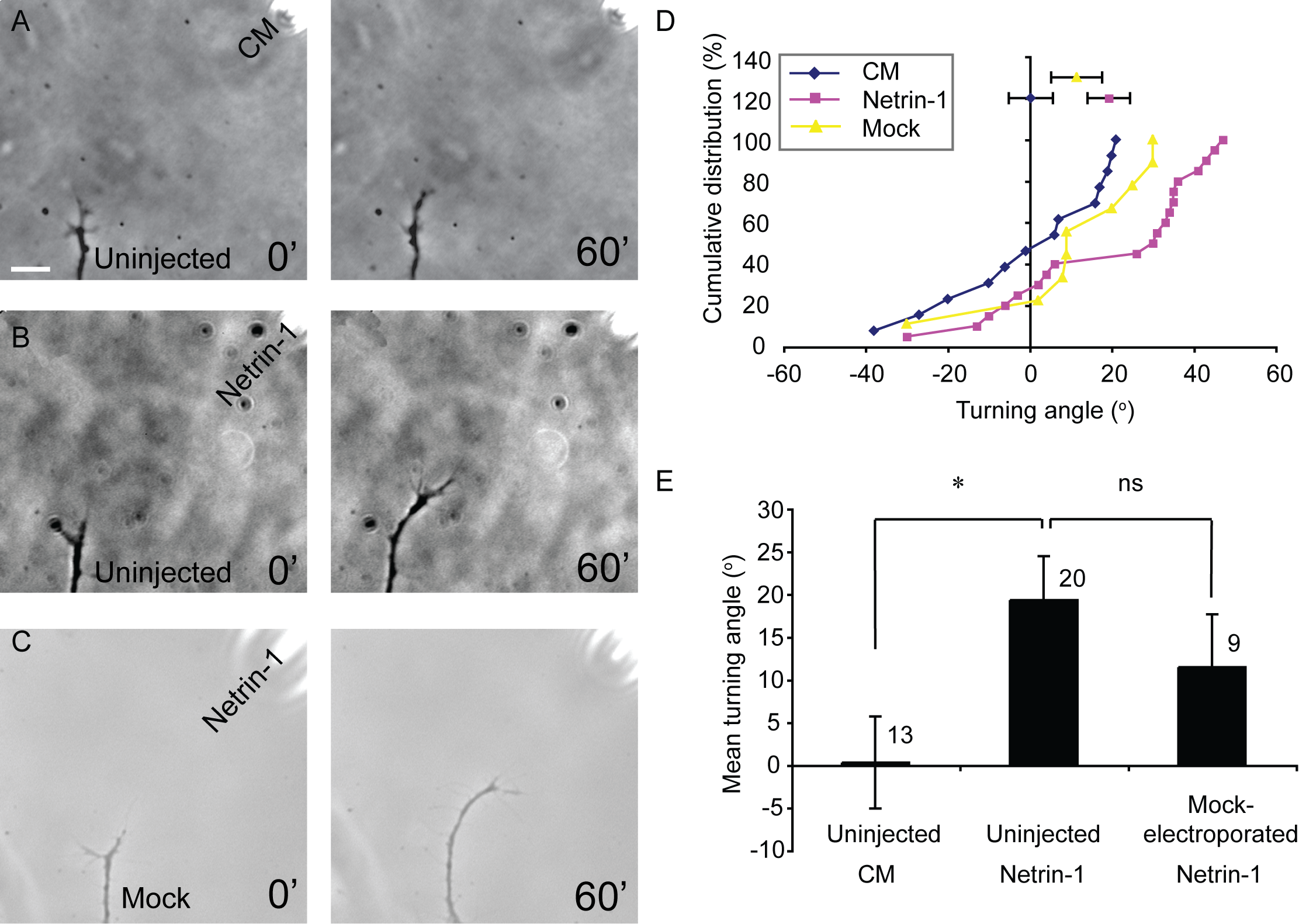

Supplement: S3 Fig — (A-C) Phase contrast images of retinal neurites from uninjected (A, B) and mock electroporated (C) retinae exposed to either a culture medium control (CM; A) or netrin-1 (B, C). (D) Cumulative distributions of turning angles of each sample group. (E) Mean turning angles of the experimental groups reveals that, whereas uninjected neurites exposed to CM did not exhibit any turning bias, neurites exposed to Netrin-1 were attracted to this guidance cue. * p < 0.05, Kolmogorov-Smirnov test. Scale bar in A: 10 μm. (TIF) [file pone.0141290.s003.tif]

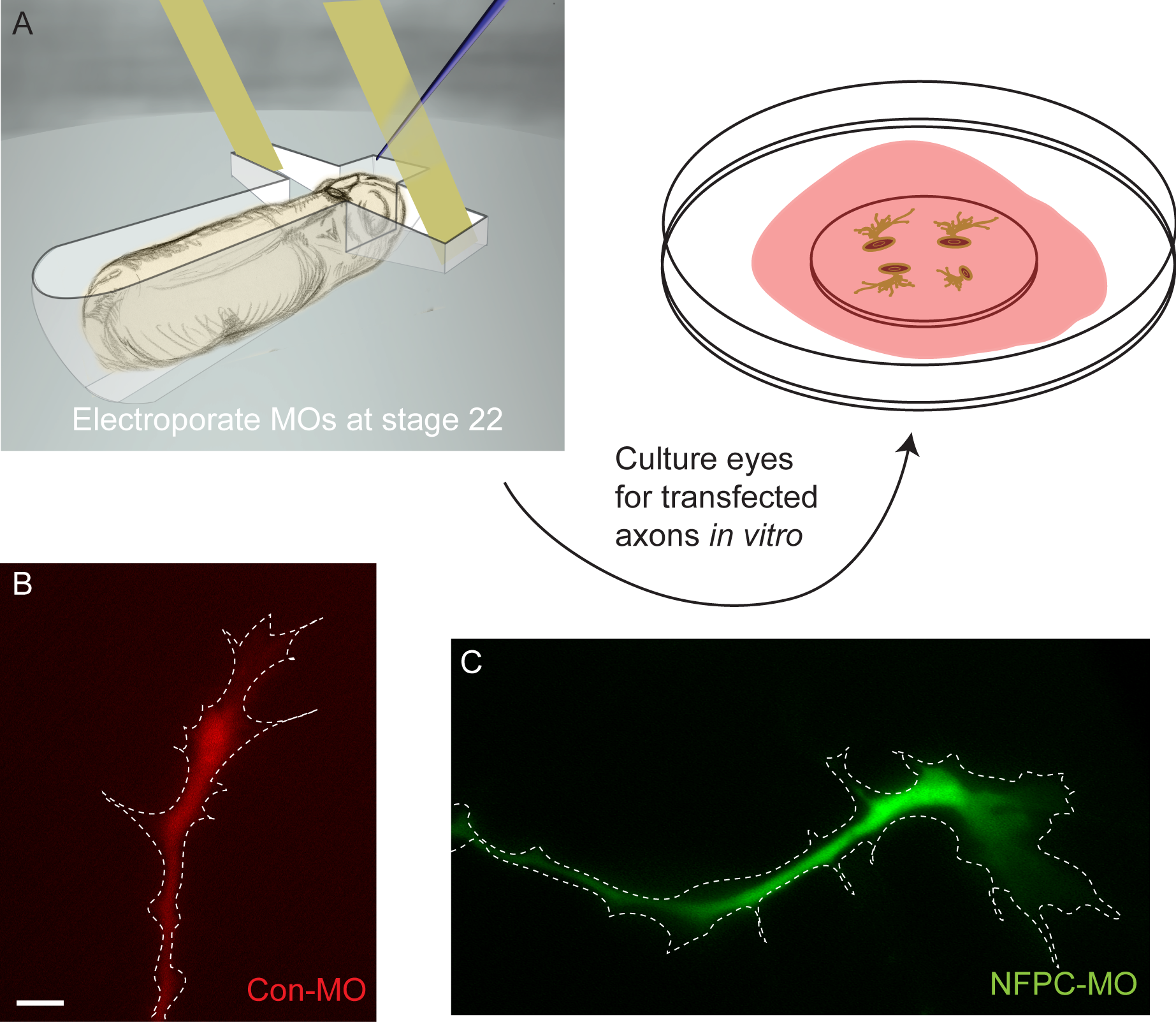

Supplement: S4 Fig — (A) Embryonic retinal primordia were electroporated with either a control, FITC-tagged morpholino (Con-MO) or a FITC-tagged anti-NFPC morpholino (NFPC-MO) using a specifically designed electroporation chamber. Eyes were then removed and cultured in vitro for 24 h. Examples of Con-MO-loaded (B, red) and NFPC-MO-loaded (C, green) neurites are shown. (TIF) [file pone.0141290.s004.tif]
